# Higher body mass index is associated with lower patient‐reported outcomes, whereas alignment and soft‐tissue balance remain comparable following imageless robotic‐assisted functional alignment total knee arthroplasty: A retrospective analysis from the Brescia Knee Research Group

**DOI:** 10.1002/jeo2.70949

**Published:** 2026-11-11

**Authors:** Guglielmo Miele, Angelo Carosini, Emanuele Sica, Lorenzo Elia, Giulio Calandra, Rudy Sangaletti, Francesco Benazzo, Luca Andriollo

**Affiliations:** ^1^ Ortopedia e Traumatologia, Università Cattolica del Sacro Cuore Rome Italy; ^2^ Brescia Knee Research Group, Fondazione Poliambulanza Istituto Ospedaliero Rome Italy; ^3^ Ospedale San Carlo di Nancy—GVM Care & Research Roma Italy; ^4^ Ortopedia e Traumatologia, Fondazione Poliambulanza Istituto Ospedaliero Brescia Italy; ^5^ Radiologia muscolo‐scheletrica, Fondazione Poliambulanza Istituto Ospedaliero Brescia Italy; ^6^ Biomedical Sciences Area, IUSS University School for Advanced Studies Pavia Italy; ^7^ Artificial Intelligence Center, Alma Mater Europaea University Vienna Austria

**Keywords:** body mass index, functional alignment, obesity, robotic surgery, total knee arthroplasty

## Abstract

**Purpose:**

Obesity is common among patients undergoing total knee arthroplasty (TKA) and may increase technical difficulty, complications and patient dissatisfaction. The purpose of this study was to evaluate the influence of body mass index (BMI) on component alignment, soft‐tissue balancing and postoperative patient‐reported outcome measures (PROMs) following primary imageless robotic‐assisted TKA performed using a functional alignment (FA) strategy.

**Methods:**

This retrospective analysis of a prospectively maintained database included 364 consecutive patients who underwent primary imageless robotic‐assisted TKA between January 2021 and December 2024 at a single high‐volume arthroplasty centre. Patients were stratified according to World Health Organization BMI classes. Radiographic alignment (mechanical hip‐knee‐ankle angle, medial proximal tibial angle, lateral distal femoral angle, posterior tibial slope), intraoperative robotic soft‐tissue laxity (final medial and lateral extension/flexion laxity in mm) and postoperative PROMs (Knee Society Score function, Forgotten Joint Score‐12 [FJS‐12], Oxford Knee Score, anterior knee pain scale, visual analogue scale pain) were compared across body mass index (BMI) categories at a mean follow‐up of 38.4 months.

**Results:**

Postoperative coronal and sagittal alignment did not differ significantly across BMI categories. Final extension and flexion gaps were comparable among groups, except for a small difference in final medial flexion laxity (*p* = 0.046), which was below the threshold of clinical relevance. Patient‐reported outcomes declined progressively with increasing BMI. The largest BMI‐related effect was observed for the FJS‐12 (*p* = 0.008), with lower scores in patients with class II and III obesity.

**Conclusions:**

In this cohort, component alignment and soft‐tissue balance after imageless robotic‐assisted TKA using an FA strategy were comparable across BMI categories, with no detectable group differences. However, higher BMI remained associated with lower PROMs, suggesting that extra‐articular, biological and biomechanical factors related to obesity may continue to influence subjective recovery even when technical precision is maintained.

**Level of Evidence:**

Level III.

**Abbreviations**
AKPS: anterior knee pain scale
BMI: body mass index
FA: functional alignment
FJS‐12: forgotten joint score‐12
IRB: Institutional Review Board
KSS: Knee Society Score
LDFA: lateral distal femoral angle
mHKA: mechanical hip‐knee‐ankle angle
MPTA: medial proximal tibial angle
OA: osteoarthritis
OKS: Oxford Knee Score
PCA: posterior condylar axis
PROM: patient‐reported outcome measure
PTS: posterior tibial slope
SD: standard deviation
TEA: transepicondylar axis
TKA: total knee arthroplasty
VAS: visual analogue scale
WHO: World Health Organization

## INTRODUCTION

Obesity, commonly defined as a body mass index (BMI) of 30 kg/m^2^ or greater, is an established independent risk factor for both the development and progression of knee osteoarthritis (OA), and the prevalence of obesity among total knee arthroplasty (TKA) candidates exceeds that observed in the general population [27, 31]. National data from the United States indicate that approximately 60%–64% of patients undergoing primary TKA are obese or morbidly obese, compared with 31%–36% in the general population [5]. Over the past three decades, the prevalence of class II obesity (BMI 35–39.9 kg/m^2^) among patients undergoing TKA has increased by 90%, while class III obesity (BMI ≥ 40 kg/m^2^) has increased by 300% [42].

Whether a specific BMI threshold should be used as an absolute contraindication to TKA remains debated. The 2023 joint guideline from the American College of Rheumatology and the American Association of Hip and Knee Surgeons recommends proceeding with TKA without delay for patients with BMI 35–39 kg/m^2^, discourages rigid BMI thresholds as absolute barriers to surgery and emphasises shared decision‐making within a multidisciplinary team [14]. Overall, current evidence does not support a universally applicable BMI cutoff for surgical eligibility, favouring instead a personalised approach that considers BMI alongside comorbidities, functional limitation and opportunities for preoperative optimisation [3, 18].

Obesity may increase the technical difficulty of TKA, particularly with respect to exposure, identification of anatomical landmarks, component positioning and soft‐tissue balancing [29]. Robotic‐assisted TKA has been proposed as a strategy to improve implant positioning and intraoperative soft‐tissue assessment through real‐time feedback and accurate bone preparation [23, 41]. Robotic platforms may also facilitate personalised alignment strategies, including functional alignment (FA), which aims to optimise three‐dimensional component positioning and soft‐tissue balance while respecting constitutional anatomy [1, 2, 7, 22]. These features may be particularly relevant in patients with obesity, in whom conventional instrumentation may be challenged by a larger soft‐tissue envelope and less reliable surface landmarks [8, 40]. Although obesity has been associated with higher rates of revision, complications, superficial and deep infection and readmission after TKA, evidence remains limited regarding the influence of BMI on intraoperative robotic balancing, alignment restoration and patient‐reported outcome measures (PROMs) after robotic‐assisted TKA performed using FA principles [33]. Recent evidence indicates that high BMI does not adversely affect radiologic component positioning or functional recovery following robotic‐assisted TKA, demonstrating the consistent precision of robotic platforms across diverse patient morphotypes [26, 36].

Despite the potential advantages of robotic assistance, it remains unclear whether accurate component positioning and balanced soft tissues can be consistently achieved across BMI categories. This question is particularly relevant because obesity may increase the technical difficulty of TKA by limiting surgical exposure, obscuring anatomical landmarks and complicating intraoperative ligament assessment. Determining whether robotic FA can overcome these challenges may help distinguish obesity‐related technical limitations from the broader biological and functional factors that influence postoperative recovery. It was hypothesised that imageless robotic TKA combined with FA would compensate for obesity‐related technical challenges by providing real‐time dynamic gap assessment and submillimetric bone resections independent of surface visibility, thereby allowing reproducible component positioning and balanced gaps regardless of patient habitus. Establishing whether robotic FA successfully preserves surgical accuracy across BMI strata guides clinical decision‐making, shifting the focus toward systemic and extra‐articular optimisation when technical precision is assured. Therefore, the purpose of this study was to evaluate the impact of BMI on alignment, robotic balancing and PROMs following imageless robotic FA‐TKA.

## METHODS

This study was conducted in accordance with the Declaration of Helsinki and approved by the local Institutional Review Board (IRB Approval No. NK5022). Written informed consent was obtained from all participants.

This retrospective analysis of a prospectively maintained institutional database included all consecutive patients who underwent primary robotic‐assisted TKA between January 2021 and December 2024 at a single high‐volume arthroplasty centre. All procedures were performed using the imageless Robotic Surgery Assistant (ROSA) Knee System (Zimmer Biomet) according to an FA philosophy.

No a priori sample size calculation was performed because this was a retrospective analysis of all consecutive eligible patients treated during the study period. Therefore, the sample size was determined by the number of patients meeting the inclusion criteria.

Patients were stratified according to the World Health Organization (WHO) BMI classification into five groups: normal weight (BMI < 25 kg/m^2^), overweight (BMI 25–29.9 kg/m^2^), obesity class I (BMI 30–34.9 kg/m^2^), obesity class II (BMI 35–39.9 kg/m^2^) and obesity class III (BMI ≥ 40 kg/m^2^). Patients undergoing revision TKA, patients with inflammatory arthritis, and procedures with incomplete demographic, robotic or radiographic data were excluded from the final analysis.

### Demographic variables included age, sex, operated side and BMI

Radiographic coronal assessment was based on long‐leg weight‐bearing radiographs and included the mechanical hip–knee–ankle angle (mHKA), medial proximal tibial angle (MPTA) and lateral distal femoral angle (LDFA) [20]. mHKA described the overall mechanical limb axis, whereas MPTA and LDFA characterised the tibial and femoral contributions to coronal alignment, respectively. Sagittal measurements were obtained from standardised lateral knee radiographs to determine the posterior tibial slope (PTS). To guarantee measurement validity and eliminate projection bias, strict true lateral alignment criteria were enforced, accepting only radiographs with a posterior condylar overlap discrepancy <3.0 mm. Measurements were recorded to the nearest 0.1 mm and 0.1° using calibrated PACS‐integrated digital measurement tools (Carestream Health/Merge Healthcare), based on embedded radiographic scaling verified against a 25.0 mm radiopaque sphere placed at the joint level or known implant dimensions. All radiographic measurements were independently performed by two trained observers blinded to clinical data and each other's evaluations, with values recorded to the nearest 0.5°. Intra and interobserver reliability were evaluated on a random sample of 50 patients, demonstrating excellent agreement, with ICC values of 0.93 (95% CI, 0.89–0.96) and 0.90 (95% CI, 0.85–0.94), respectively.

Intraoperative robotic data were extracted from the ROSA system and included initial medial and lateral laxity in extension and flexion, planned tibial slope and coronal alignment, planned tibial and femoral resections, femoral rotational alignment relative to the posterior condylar axis (PCA) and trans epicondylar axis (TEA), and final extension and flexion balancing.

PROMs were collected at the latest follow‐up and included the Knee Society Score function sub score (KSS function), forgotten joint score‐12 (FJS‐12), Oxford Knee Score (OKS), Kujala anterior knee pain scale (AKPS), Visual Analogue Scale for pain (VAS pain) and a Likert satisfaction scale [6, 15, 32, 34, 38].

All procedures were performed using the imageless ROSA Knee System (Zimmer Biomet) in combination with the Persona Knee System (Zimmer Biomet) using a standardised FA philosophy combined with a tibia‐first workflow [22, 23]. Target mHKA was individualised according to constitutional alignment, maintained within 174°–180° for varus/neutral knees and 177°–183° for valgus knees. Component orientations were maintained within safety boundaries: LDFA between 84° and 93°, MPTA between 84° and 92° and femoral flexion between 2° and 4°. Initial target PTS was 5° for medial congruent (MC) inserts and 3° for posterior‐stabilised (PS) inserts (±1° adjustment allowance).

Following anatomical landmark acquisition, the proximal tibial cut was performed first. Ligament balancing was then dynamically assessed using the FuZion tensioning device (Zimmer Biomet) in extension and at 90° of flexion. Distal femoral resection and axial femoral rotation were dynamically adjusted according to soft‐tissue behaviour [23, 40]. Planned gap targets were set at 19 mm in extension and 19 mm (MC) or 20 mm (PS) in flexion. Final robotic balancing parameters recorded from the ROSA platform included final medial and lateral laxity in extension and flexion (in mm), targeting 0–1 mm of residual laxity in extension and 0–1.5 mm in flexion (with up to 1 mm additional lateral compartment laxity considered acceptable for MC inserts). Soft‐tissue releases were avoided unless the target balance could not be achieved within the predefined osseous adjustment limits. Patellar resurfacing was performed routinely in all patients. All procedures were performed by three senior fellowship‐trained arthroplasty surgeons using the exact same standardised imageless robotic workflow and FA targets.

### Statistical analysis

Continuous variables were expressed as mean ± standard deviation (SD), while categorical variables were reported as frequencies and percentages. Normality was assessed using the Shapiro–Wilk test. Comparisons among BMI classes were performed using a one‐way analysis of variance (ANOVA) for normally distributed continuous variables, whereas nonnormally distributed variables were analysed using the Kruskal–Wallis *H‐* test. Pairwise post hoc analyses were adjusted using the Holm correction method. Categorical variables were compared using chi‐square or Fisher exact tests. Correlations between BMI and radiographic parameters (mHKA, MPTA, LDFA and PTS), robotic soft‐tissue laxity parameters (medial and lateral laxity in extension and flexion) and PROMs (KSS function, FJS‐12, OKS, AKPS, VAS pain and Likert satisfaction scale) were assessed using Spearman correlation coefficients. Statistical significance was set at *p* < 0.05 with a 95% confidence interval. A post hoc power analysis was performed using the FJS‐12 as a key patient‐reported outcome. Based on the observed group means, SDs and sample sizes, the estimated effect size was Cohen's *f* = 0.294. With five BMI categories, a total sample size of 364 patients and an alpha level of 0.05, the observed power for the overall comparison was 99.8%. Statistical analyses were performed using Python version 3.11.

## RESULTS

### Study population

A total of 364 patients with available BMI data were included in the final analysis (Table 1).

**Table 1:** Demographic characteristics of the study population according to the World Health Organization body mass index (BMI) classification.

Variable	Normal weight	Overweight	Obesity I	Obesity II	Obesity III	*p*‐value
Patients, *n*	136	137	62	21	8	‐
Age (years)	68.8 ± 10.5	68.9 ± 9.8	69.4 ± 9.1	70.0 ± 8.8	67.0 ± 12.6	0.765
Female sex (%)	51.5	53.3	59.7	61.9	62.5	0.441

*Note*: Data are presented as mean ± standard deviation or number of patients (%), as appropriate.

Overall, the cohort comprised 198 female patients (54.4%) and 166 male patients (45.6%).

According to the WHO BMI classification, 136 patients (37.4%) were normal weight, 137 (37.6%) were overweight, 62 (17.0%) had class I obesity, 21 (5.8%) had class II obesity and 8 (2.2%) had class III obesity. Mean age was comparable among groups (*p* = 0.765), ranging from 67.0 ± 12.6 years in patients with class III obesity to 70.0 ± 8.8 years in patients with class II obesity. The proportion of female patients increased with BMI category, although the difference was not statistically significant (*p* = 0.441). Mean follow‐up was 38.4 ± 13.9 months (range, 15.3–62.5 months).

### Radiographic outcomes

Preoperative radiographic alignment did not differ significantly among BMI categories (Table 2), establishing comparable baseline parameters across all study groups before evaluating postoperative outcomes. Preoperative mHKA ranged from 176.0° to 178.3° across groups (*p* = 0.726). No significant differences were observed for MPTA (*p* = 0.534), LDFA (*p* = 0.563) or PTS (*p* = 0.628).

**Table 2:** Preoperative radiographic parameters according to body mass index class.

Variable	Normal weight	Overweight	Obesity I	Obesity II	Obesity III	*p*‐value
mHKA (deg)	176.8 ± 8.1	176.0 ± 7.8	177.1 ± 8.0	177.5 ± 7.2	178.3 ± 4.6	0.726
MPTA (deg)	88.3 ± 4.0	87.6 ± 3.7	87.7 ± 3.9	87.3 ± 3.5	87.2 ± 2.1	0.534
LDFA (deg)	88.6 ± 3.4	88.8 ± 3.7	88.1 ± 3.3	88.0 ± 3.5	86.7 ± 3.1	0.563
Posterior tibial slope (deg)	6.5 ± 4.4	6.0 ± 4.0	6.1 ± 4.3	5.5 ± 3.9	2.9 ± 3.8	0.628

*Note*: Data are presented as mean ± standard deviation.

Abbreviations: LDFA, lateral distal femoral angle; mHKA, mechanical hip–knee–ankle angle; MPTA, medial proximal tibial angle.

Postoperative alignment restoration was comparable across BMI categories: postoperative mHKA was $177.3^{\circ }\pm 2.7^{\circ }$ in normal weight, $177.1^{\circ }\pm 2.7^{\circ }$ in overweight, $177.5^{\circ }\pm 3.4^{\circ }$ in class I, $178.7^{\circ }\pm 3.9^{\circ }$ in class II and $177.0^{\circ }\pm 1.7^{\circ }$ in class III obesity (p=0.400) (Table 3). Postoperative MPTA, LDFA and PTS were also comparable among BMI classes (all *p* > 0.05). Overall, robotic‐assisted TKA achieved consistent restoration of coronal and sagittal alignment regardless of BMI category.

**Table 3:** Postoperative radiographic parameters according to body mass index class.

Variable	Normal weight	Overweight	Obesity I	Obesity II	Obesity III	*p*‐value
mHKA (deg)	177.3 ± 2.7	177.1 ± 2.7	177.5 ± 3.4	178.7 ± 3.9	177.0 ± 1.7	0.400
MPTA (deg)	89.3 ± 2.1	88.9 ± 1.9	89.0 ± 2.0	88.8 ± 1.6	88.7 ± 0.2	0.670
LDFA (deg)	90.4 ± 2.2	90.4 ± 1.8	90.6 ± 1.8	89.3 ± 2.6	90.5 ± 1.0	0.363
Posterior tibial slope (deg)	2.7 ± 1.3	2.4 ± 1.6	2.8 ± 2.1	2.4 ± 1.7	1.0 ± 0.9	0.291

*Note*: Data are presented as mean ± standard deviation.

Abbreviations: LDFA, lateral distal femoral angle; mHKA, mechanical hip–knee–ankle angle; MPTA, medial proximal tibial angle.

### Intraoperative robotic balancing

Robotic intraoperative balancing demonstrated comparable results across BMI categories (Table 4). Final extension and flexion laxity values were similar among groups, with no progressive increase in gap asymmetry or instability as BMI increased. The only statistically significant difference was observed for final medial flexion laxity (*p* = 0.046).

**Table 4:** Final intraoperative robotic soft‐tissue balancing parameters according to body mass index class.

Variable	Normal weight	Overweight	Obesity I	Obesity II	Obesity III	*p*‐value
Final extension medial laxity (mm)	2.6 ± 1.3	2.5 ± 1.2	2.4 ± 1.1	2.3 ± 1.0	2.2 ± 0.9	0.418
Final extension lateral laxity (mm)	2.8 ± 1.6	2.7 ± 1.4	2.5 ± 1.2	2.4 ± 1.1	2.4 ± 1.0	0.377
Final flexion medial laxity (mm)	1.8 ± 0.9	1.7 ± 0.9	1.5 ± 0.8	1.5 ± 0.7	1.4 ± 0.7	0.046
Final flexion lateral laxity (mm)	2.2 ± 1.3	2.1 ± 1.2	2.0 ± 1.0	2.0 ± 0.9	1.9 ± 0.8	0.430

*Note*: Data are presented as mean ± standard deviation. Laxity values are expressed in millimetres.

Post hoc pairwise analysis for final medial flexion laxity demonstrated a significant difference between normal‐weight patients and patients with class I obesity (mean difference, 0.3 mm; adjusted *p* = 0.041). Although this difference reached statistical significance, its absolute magnitude was only 0.3 mm and is unlikely to be clinically meaningful. No other pairwise comparisons remained significant after Holm correction.

### Clinical outcomes

Postoperative PROMs were available for all 364 patients and are summarised in Table 5. A progressive reduction in PROMs was observed across increasing BMI category, particularly among patients with class II and III obesity. However, as these univariable comparisons do not account for potential confounding variables, these findings represent unadjusted associations and should be interpreted with appropriate caution. FJS‐12 demonstrated the most pronounced BMI‐related effect (*p* = 0.008), from 89.1±18.4 in normal weight and 87.3±19.1 in overweight patients to 72.5±17.8 in class II and 64.2±15.3 in class III obesity (p=0.008); however, given the small sample size of the class III obesity subgroup (n=8), these estimates should be interpreted cautiously as exploratory due to limited statistical power. Similarly, KSS function decreased from 89.4±15.8 in normal weight to 71.2±12.5 in class III obesity (p=0.031). AKPS values also declined with increasing BMI (*p* = 0.027), suggesting worse patellofemoral‐related function in patients with higher BMI. OKS and VAS pain showed nonsignificant trends toward worse outcomes with increasing BMI.

**Table 5:** Postoperative outcomes according to body mass index class.

Variable	Normal weight	Overweight	Obesity I	Obesity II	Obesity III	*p*‐value
KSS function	89.4 ± 15.8	87.9 ± 16.2	83.1 ± 15.4	78.6 ± 14.9	71.2 ± 12.5	0.031
FJS‐12	89.1 ± 18.4	87.3 ± 19.1	80.4 ± 18.9	72.5 ± 17.8	64.2 ± 15.3	0.008
OKS	42.8 ± 7.4	42.1 ± 8.1	40.7 ± 7.3	39.2 ± 6.8	37.8 ± 6.4	0.114
AKPS	82.1 ± 13.8	80.6 ± 14.1	76.8 ± 14.9	71.5 ± 13.7	68.1 ± 12.6	0.027
VAS pain	8.2 ± 14.5	9.5 ± 16.1	11.6 ± 17.9	14.9 ± 18.7	17.2 ± 20.4	0.091

*Note*: Data are presented as mean ± standard deviation.

Abbreviations: AKPS, anterior knee pain scale; FJS‐12, forgotten joint score‐12; KSS, Knee Society Score; OKS, Oxford knee core; SD, standard deviation; VAS, visual analogue scale—VAS, 100‐mm visual analogue scale.

## DISCUSSION

The main findings of this study are that robotic‐assisted FA TKA achieved comparable radiographic alignment and intraoperative soft‐tissue balance across BMI categories, whereas PROMs progressively worsened with increasing BMI, particularly for the FJS‐12. This study aimed to evaluate the impact of BMI on alignment, soft‐tissue balance and PROMs following imageless robotic‐assisted FA‐TKA. It was hypothesised that robotic FA would achieve comparable component alignment and soft‐tissue balance across BMI categories. Confirming our hypothesis, coronal and sagittal alignment as well as intraoperative gap balancing were consistent across all BMI groups, with only a clinically negligible 0.3 mm difference in final medial flexion laxity. When evaluating PROMs across BMI categories, scores progressively declined with increasing BMI, most prominently for FJS‐12. Our radiographic evaluation confirmed that robotic‐assisted TKA using a FA strategy achieved accurate and highly reproducible component positioning across all BMI categories. Coronal parameters, including overall limb axis (mHKA), MPTA and LDFA, showed no significant differences among BMI classes, and sagittal alignment (PTS) remained consistently within planned parameters across all groups. In conventional TKA, excess adiposity can obscure external bony landmarks, thereby increasing the rate of coronal and sagittal positioning outliers. Our findings suggest that imageless robotic registration may help to overcome these physical constraints by relying on intraoperative anatomic registration rather than surface landmarks, providing stable alignment parameters regardless of patient morphotype. In addition to component alignment, robotic assistance allows for immediate real‐time evaluation of medial and lateral gaps both in flexion and extension. By providing dynamic intraoperative assessment of bone resections, component positioning and ligament balance, robotic platforms can standardise soft‐tissue evaluation and may enhance surgical decision‐making compared with conventional techniques [40, 41]. This real‐time feedback facilitates reproducible execution even in technically demanding patients in whom excess soft tissue or adiposity may obscure traditional anatomical landmarks [8, 40, 41]. In our cohort, final gaps were symmetric across all BMI categories with only a clinically negligible 0.3 mm difference in final medial flexion laxity, supporting the concept that a robot‐guided FA workflow can mitigate obesity‐related technical challenges while preserving precise alignment control [1, 10]. These comparable radiographic and balancing results are consistent with previous studies suggesting that robotic assistance improves component positioning accuracy and reduces alignment outliers, producing narrower error distributions across diverse morphotypes [8, 9, 19, 43]. Ultimately, the present study extends these observations by illustrating that an imageless robotic system integrated within an FA workflow maintains consistent component alignment and balanced flexion‐extension gaps across the entire spectrum of BMI. FA aims to restore constitutional anatomy while achieving balanced soft tissues by co‐optimising three‐dimensional bone resections and ligament tension within safe coronal boundaries [22, 23]. When executed with robotic guidance, extensive evidence indicates that FA enhances soft‐tissue balance and potentially mitigates mid‐flexion instability, thereby reducing the need for soft‐tissue releases, without compromising coronal or overall alignment accuracy [13, 28].

However, despite these excellent and consistent intraoperative and radiographic metrics, this technical precision did not directly translate into equivalent clinical outcomes across all groups, as postoperative PROMs progressively worsened with higher BMI classes.

The greatest differences were observed for the FJS‐12, though the small sample size of the Class III obesity subgroup (n=8) limits statistical power, warranting a cautious interpretation. Together, these findings suggest that robotic‐assisted FA‐TKA may help mitigate the technical challenges associated with obesity. The progressive decline in PROMs across increasing BMI categories observed in this study is consistent with previous reports showing lower subjective outcomes and greater residual symptoms among patients with obesity after TKA [4, 11, 12, 16, 37]. Importantly, the present study indicates that these differences persist despite comparable implant positioning and soft‐tissue balance. This suggests that the relationship between obesity and postoperative recovery extends beyond surgical accuracy alone. Similarly, in a retrospective cohort study, McCormick et al. found no additional functional benefit of robotic‐assisted TKA in obese patients [30]. Although most available evidence on the relationship between TKA outcomes and obesity is derived from mechanically aligned implants, there is currently no clear evidence as to whether robotic‐assisted TKA performed with FA principles leads to superior clinical outcomes in obese patients. In a recent commentary, Pegreffi et al. emphasised that conditions such as sarcopenia and impaired muscle status may influence functional recovery after TKA independently of implant alignment [35]. While unmeasured factors, such as elevated joint loading, reduced physical conditioning, chronic low‐grade inflammation, metabolic dysfunction, psychosocial parameters or altered movement patterns, have been hypothesised in the literature to influence outcomes, these variables were not directly evaluated in our cohort [17]. Therefore, their specific role in our study population remains speculative. Overall, our findings suggest that optimal implant positioning alone may not fully overcome the broader biological and biomechanical burden associated with obesity. These findings have practical implications. While our data confirm that high BMI alone should not preclude patients from undergoing TKA due to alignment or balancing technical failure, the lower PROMs observed in higher BMI categories highlight the complex, multifactorial nature of postoperative outcomes. In line with current literature, factors such as prehabilitation, metabolic control, nutritional support and targeted weight‐loss strategies have been proposed to address extra‐articular contributors to inferior outcomes [17, 24, 25, 39]. Kokubu et al. reported in a retrospective cohort study that preoperative nutritional status was associated with long‐term functional outcomes after TKA, supporting a comprehensive assessment of the patient's clinical condition beyond BMI alone [21]. However, as perioperative optimisation protocols were not directly investigated in the present study, future research is required to determine their specific impact and optimal timing relative to TKA in this challenging population.

The strengths of this study include the analysis of a large consecutive cohort derived from a prospectively maintained database, the use of a standardised imageless robotic platform with a consistent FA workflow, and the availability of objective intraoperative balancing data. However, several limitations should be acknowledged. First, the retrospective single‐centre design introduces the possibility of selection bias and may limit the generalisability of our findings. Although the study included all consecutive eligible patients from a prospectively maintained institutional database, treatment was not randomly allocated, and the study population reflects the referral patterns, surgical indications and perioperative protocols of a single high‐volume arthroplasty centre. Consequently, our findings may not be directly applicable to lower‐volume institutions or different healthcare settings. Second, the distribution of patients across BMI categories was substantially unbalanced, with relatively small numbers of patients in Class II (n=21) and Class III (n=8) obesity. While these numbers reflect the natural epidemiology of our patient population, these small subgroup sizes limit statistical power and preclude definitive conclusions for morbidly obese patients. Consequently, findings concerning high‐BMI subgroups should be considered exploratory and warrant confirmation in larger, dedicated cohorts. Third, because no a priori sample size calculation was performed, the study may have been underpowered to detect small differences between BMI groups, particularly for the less represented obesity classes. Therefore, the absence of statistically significant differences for radiographic and robotic balancing parameters should not be interpreted as definitive evidence of the absence of an effect. Larger multicentre studies are warranted to confirm these findings. Fourth, although all patients achieved a minimum follow‐up exceeding 1 year and the overall mean follow‐up was 38.4 months, follow‐up duration ranged from 15.3 to 62.5 months, which may introduce minor variation in mid‐term clinical evaluations. Fifth, preoperative PROMs and detailed comorbidity data (e.g., ASA score, diabetes status, baseline functional level) were unavailable in our institutional database. Consequently, multivariable regression analyses could not be performed to adjust for potential confounders, limiting our ability to isolate the independent effect of BMI on PROMs. The observed differences in PROMs across BMI categories must therefore be interpreted as unadjusted associations subject to residual confounding. Sixth, BMI does not distinguish between adiposity and lean body mass or account for fat distribution. Additionally, this study is limited by the absence of long‐term data regarding implant survival and the lack of a defined revision endpoint. Furthermore, we did not include a dedicated complication analysis, such as the rate of readmissions, medical events or reoperations, which represents a critical component when evaluating outcomes in high‐BMI cohorts.

Finally, although a statistically significant difference in final medial flexion laxity was observed between normal‐weight patients and patients with class I obesity, the absolute difference was only 0.3 mm. Statistical significance should not be interpreted as synonymous with clinical relevance, particularly when differences are smaller than the reported measurement accuracy of contemporary robotic systems. Furthermore, no validated minimal clinically important difference has been established for intraoperative robotic gap measurements; therefore, this finding should be interpreted with caution. Future studies with extended follow‐up are needed to assess whether the short‐term technical accuracy provided by robotic FA translates into durable implant survivorship and reduced complication rates in this population.

## CONCLUSIONS

Robotic‐assisted FA TKA achieves consistent precision and soft‐tissue balance across all BMI categories. However, higher BMI remains associated with inferior patient‐reported outcomes, underscoring that surgical accuracy alone does not fully drive postoperative recovery in severe obesity.

## AUTHOR CONTRIBUTIONS

Luca Andriollo and Guglielmo Miele had the idea for the article. Luca Andriollo was responsible for writing the manuscript and qualified as the corresponding author. Lorenzo Elia, Giulio Calandra, Emanuele Sica and Emanuele Sica were responsible for data acquisition. Luca Andriollo was responsible for statistical analysis. Luca Andriollo and Emanuele Sica were responsible for conceptualisation and supervised data acquisition and analysis. Emanuele Sica, Angelo Carosini and Angelo Carosini were responsible for reviewing and critically revise the manuscript. All authors have given final approval of the version to be published.

## FUNDING INFORMATION

The authors have no funding to report.

## CONFLICT OF INTEREST STATEMENT

Guglielmo Miele, Angelo Carosini, Emanuele Sica, Lorenzo Elia, Giulio Calandra, Rudy Sangaletti and Luca Andriollo have nothing to declare. Francesco Benazzo has a consulting contract with Zimmer Biomet.

## ETHICS STATEMENT

This study was conducted in accordance with the Declaration of Helsinki and approved by the local Institutional Review Board (IRB Approval No. NK5022). Written informed consent was obtained from all participants. All patients provided legitimate informed consent.

## Data Availability

The data that support the findings of this study are available from the corresponding author, [Luca Andriollo], upon reasonable request.
